# A Case Report of Invasive Mucormycosis in a COVID-19 Positive and Newly-Diagnosed Diabetic Patient

**DOI:** 10.21980/J81M1G

**Published:** 2023-07-31

**Authors:** Konnor Davis, Roy Almog, Yuval Peleg, Lindsey Spiegelman

**Affiliations:** * University of California, Irvine, School of Medicine, Irvine, CA; ^ University of California, Irvine, Department of Emergency Medicine, Orange, CA

## Abstract

This is a case report of a 46-year-old female with undiagnosed diabetes, COVID-19, and mucormycosis. Mucormycosis is a rare, rapidly progressive disease process characterized by an insidious onset of vague symptoms and is associated with a high mortality rate. The patient initially presented at two outside ophthalmology clinics due to right eye pain and was prescribed steroids. Upon presentation to our institution’s Emergency Department, the patient’s pain had significantly increased along with new symptoms of ptosis and failure to adduct the right eye. Laboratory results demonstrated leukocytosis, hyperglycemia, and a positive SARS-CoV-2 test. Magnetic resonance imaging confirmed a diagnosis of mucormycosis and the patient underwent surgery and began intravenous antifungal therapy. This discussion addresses the presenting features of mucormycosis in the emergency department while highlighting the need for immediate investigation due to the fungi’s rapidly progressive nature.

**Topics:**

Mucormycosis, mucor, diabetes, COVID-19, ROCM.

[Fig f1-jetem-8-3-v10] [Fig f2-jetem-8-3-v10] [Fig f3-jetem-8-3-v10]

**Figure f1-jetem-8-3-v10:**
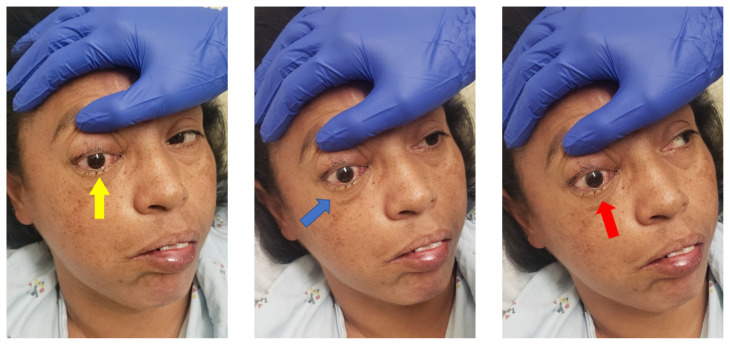
Eye Exam Video Link: https://youtu.be/ziINu35zev4

**Figure f2-jetem-8-3-v10:**
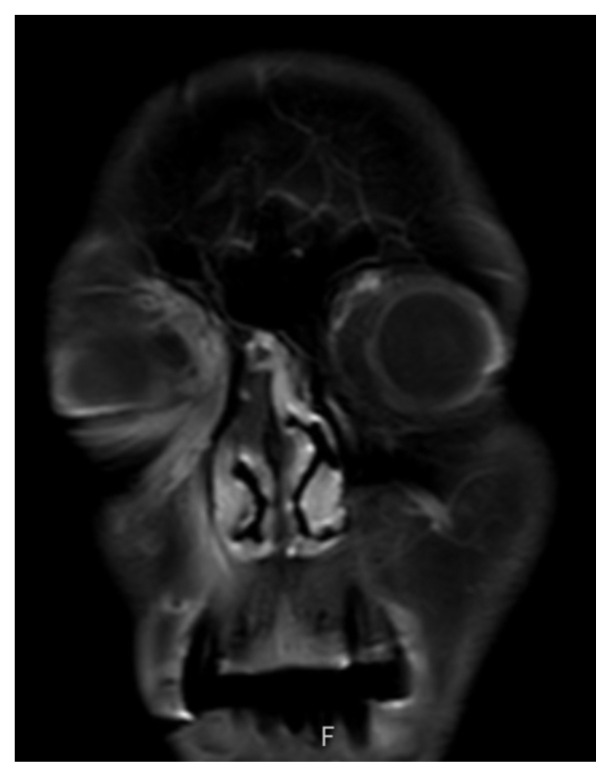


**Figure f3-jetem-8-3-v10:**
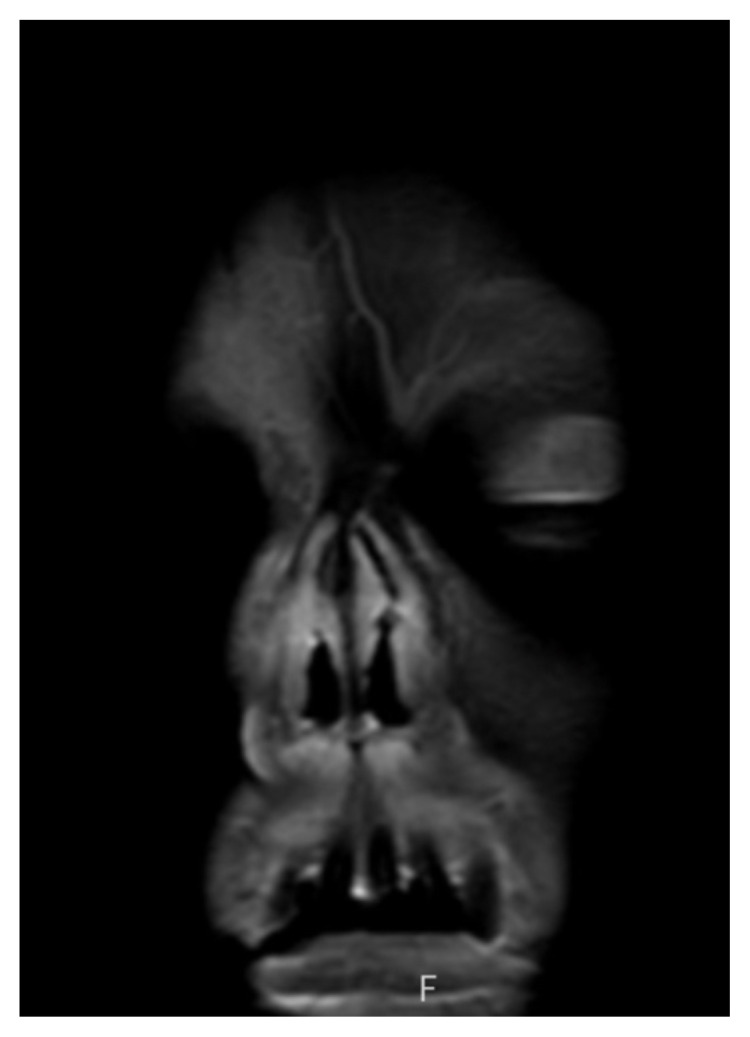


## Brief introduction

Mucormycosis (colloquially shortened to “mucor”) is a rapidly progressing fungal infection associated with high morbidity and mortality from molds within the order Mucorales.^1^ In developed countries such as the United States, mucormycosis infections occur primarily in immunocompromised individuals. In addition, uncontrolled diabetes mellitus is an important risk factor.^2^ Mucormycosis infection and clinical presentation is dependent on anatomic location, with the most common site of involvement being the sinuses (a condition called rhino-orbital-cerebral mucormycosis, ROCM) that presents as nonspecific signs and symptoms like fever and headache or more specific instances of dark nasal or palatine mucosal eschar, orbital invasion, and brain involvement.^2^ Although the condition is rare, it is a surgical and medical emergency due to the fungi’s propensity for angioinvasion and brain involvement. Here we describe an unusual case of rapidly deteriorating mucormycosis in a patient with undiagnosed diabetes and COVID-19.

## Presenting concerns and clinical findings

A 46-year-old female with no known prior medical history presented to the emergency department (ED) with one week of constant and sharp right eye pain. The patient had previously seen two ophthalmologists and was given steroid eye drops but presented to our ED for continued pain. On presentation the patient was febrile, hypertensive, and endorsed headaches, chest pain, shortness of breath, and vomiting. Upon physical examination, there was tenderness, conjunctival injection, restricted ocular range of motion (lack of lateral motion in both adduction and abduction), and ptosis of the right eye. Written consent was obtained from patient for photograph and image publication.

## Significant findings

On physical exam, when the patient was asked to try and look to her right, the right eye failed to move laterally/abduct (blue arrow). Additionally, when asked to look straight ahead, the eye was slightly adducted (red arrow). There was a lack of motion of the right eye in abduction when the patient was asked to look to her right (yellow arrow). Labs were significant for leukocytosis (white blood cell count 21.4 **×** 10^9^/Liter), a non-fasting glucose of 385 milligrams per deciliter (mg/dL), and a positive SARS-CoV-2 test. Initial differential diagnoses included third nerve palsy (secondary to diabetes), cavernous sinus thrombosis, aneurysm, orbital cellulitis, or central retinal artery occlusion. A computed tomography angiogram (CTA) of the head and neck was done but no acute pathology was reported. Given the clinical concern from the abnormal physical exam findings, after the CTA results were obtained, both orbital and brain magnetic resonance imaging (MRI) scans (both with and without contrast) were performed in the emergency department. These indicated asymmetric enhancement of the right nasal cavity turbinates, T1 hypointense enhancing soft tissue involving the right prefrontal and prenasal soft tissues extending into the right medial extraconal orbit, and areas of restricted diffusion in the bilateral rectus gyri.

## Patient course

The patient’s MRIs provided evidence of bilateral frontal lobe cerebrovascular accident, right optic neuropathy, and concern for invasive fungal infection. The patient was then admitted to the internal medicine team with neurology, endocrinology, ophthalmology, and otolaryngology following the patient. The following day, the patient underwent right-sided medial maxillectomy, frontal sinusotomy, sphenoidotomy, total ethmoidectomy, septectomy, and resection of the pterygopalatine fossa and infratemporal fossa. The patient was started on intravenous amphotericin and micafungin in the days following surgery with subsequent hypokalemia and hypomagnesemia. Given the nature and prognosis of invasive fungal sinusitis (mucormycosis) with intracranial and right orbital extension, neurosurgery did not recommend further surgical interventions and the palliative care team was consulted regarding goals of care discussions. One month after presenting to the ED, the patient was discharged home to receive hospice care.

## Discussion

Mucormycosis is an invasive and opportunistic fungal infection with all-cause mortality rates ranging between 40% and 80% dependent on underlying conditions and where the infection originated.^1^,^3^ The incidence of mucormycosis has increased over time, due to an increased number of immunocompromised patients, but international disparities still exist.^4^ In developed countries, the most common underlying condition is hematological malignancies whereas in developing countries, the disease affects mostly diabetic or trauma patients.^4^,^5^ Previous research has pointed to a pulmonary presentation (high-grade fever and nonproductive cough) being most common in patients with hematological malignancies. However, lung involvement is less common today and the rhino-orbital-cerebral presentation must be considered as well.^4^,^5^ Due to mucor’s predilection for angioinvasion, concerns are heightened because the fungus can cause tissue infarctions, necrosis, and systemic dissemination.^5^ Specific to ROCM, which begins as the fungus is inhaled into the paranasal sinuses, patients often present with unilateral periorbital facial pain, inflammation, eyelid edema, headache, acute vision loss, and may eventually demonstrate the characteristic black necrotic eschar of the disease.^2^,^5^ Diagnostic methods include CT, MRI, endoscopy, and biopsy.^1^ Current treatment guidelines prioritize aggressive surgical and medical management with intravenous amphotericin B being the antifungal of choice.^1^,^2^,^5^

While much of the data on mucormycosis comes from case reports, case series, and autopsy reports, a clear link has been established between mucor and diabetes mellitus.^5^ Various authors have reported diabetes as a predisposing factor in anywhere from 36% to 88% of cases.^5^ Diabetes provides an example of a complex interaction between fungus and host, with patients with elevated blood glucose levels leading to impaired neutrophil function, ketoacidosis (not seen in this patient) leading to a defect in motility and killing by the neutrophils due to the acidic pH, and systemic alterations increasing the free iron available to fungal cells.^6^,^7^ Each of these cellular and systemic changes leads to a situation where mucormycosis can thrive. Similarly, yet less well understood, is the interplay between host, fungus, and COVID-19. Numerous case reports worldwide have appeared in the literature recently describing patients acquiring mucor either during or after infection with SARS-CoV-2, including many with underlying diabetes as well.^6^–^10^ Concerns for mucormycosis specific to patients with COVID-19 include immune dysregulation (mainly T lymphocytes), administration of corticosteroids that can magnify hyperglycemia, intubation providing a route for spore inhalation, and cytokine release increasing serum iron.^8^,^11^,^12^ Physicians are aware of the major effects and complications of COVID-19, including immunosuppression, but should also be sure to include mucormycosis in their differential of patients with COVID-19 and complicated rhinosinusitis cases.^8^ Overall, there are many conditions that predispose individuals to mucormycosis, including diabetes, hematological malignancies, immunocompromise, corticosteroid use, and COVID-19.

This case demonstrates a rare fungal infection that is important for medical providers to urgently recognize and understand. The angioinvasive fungal infection leads to rhinocerebral, cutaneous, pulmonary, and/or system signs and symptoms that are associated with rapid progression, morbidity, and mortality.^1^ A thorough past medical history can help elucidate if there are predisposing conditions to this disease. Early CT and MRI imaging, as obtained in this case, are essential in making this diagnosis in order to immediately begin antifungal treatment and/or undergo major surgical resection of infected tissue. Consultation and coordination with various specialists are often necessary to manage the care of these high-risk patients. Ultimately, palliative care teams may be necessary to consult for discussion of treatment course, goals of care, and end of life discussions.

## Supplementary Information


















