# Supplementary figures and images for: A Case Report of Invasive Mucormycosis in a COVID-19 Positive and Newly-Diagnosed Diabetic Patient
Source: J Educ Teach Emerg Med. 2023 Jul 31;8(3):V10–3. doi: 10.21980/J81M1G (PMC10414977; doi:10.21980/J81M1G)

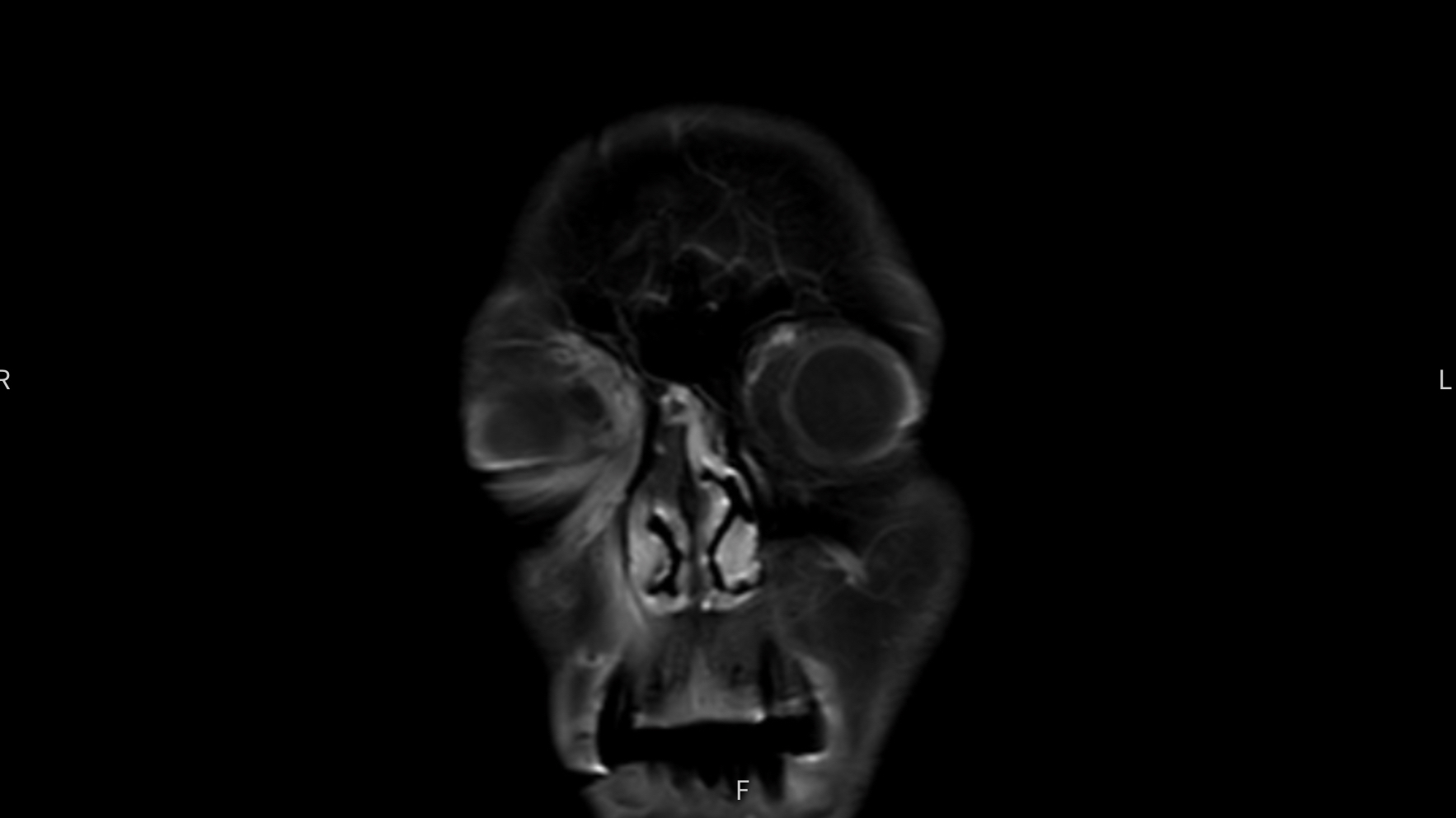

Supplement: Supplementary file 1 [file JETem-8-3-V10-supp1.jpg]

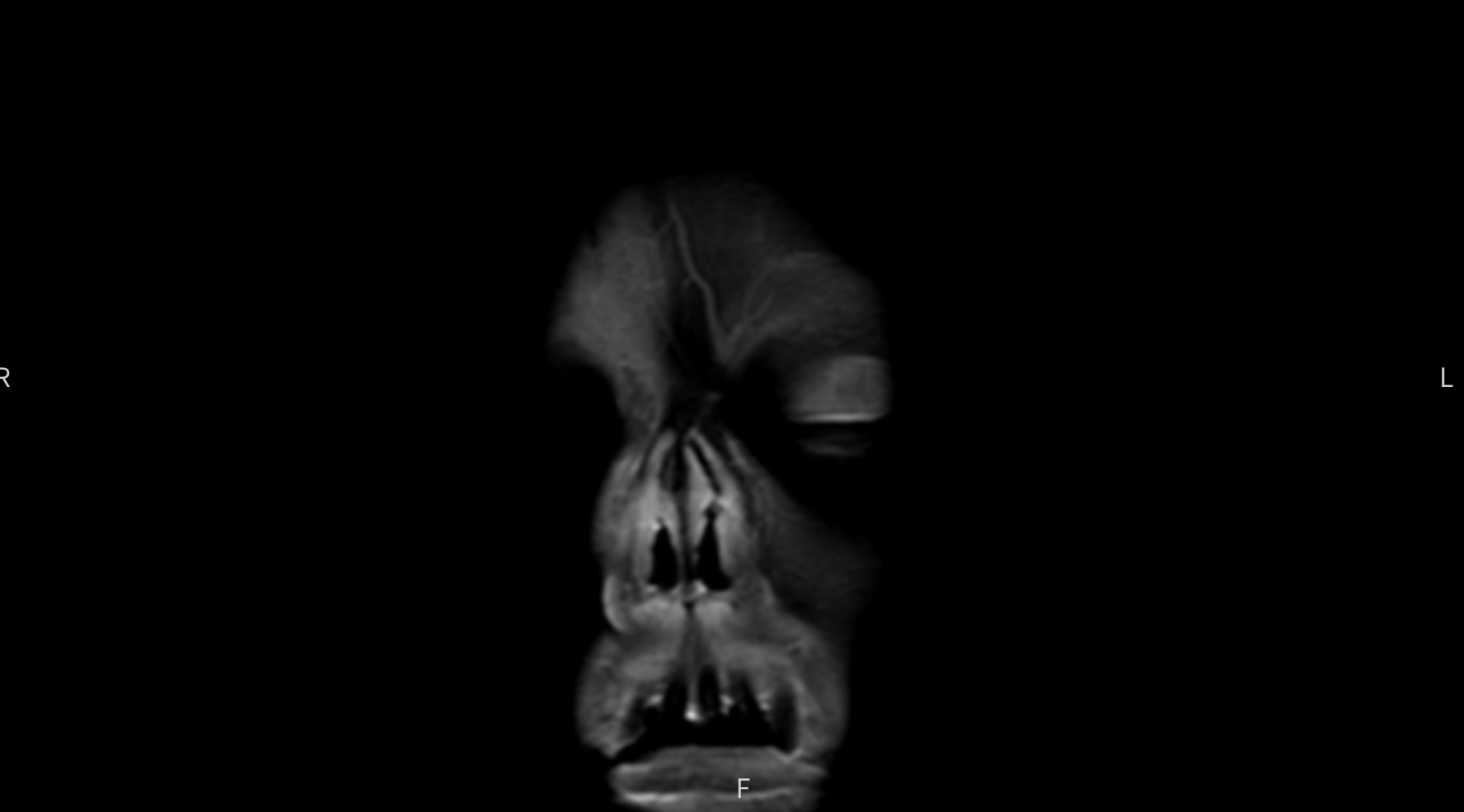

Supplement: Supplementary file 2 [file JETem-8-3-V10-supp2.jpg]

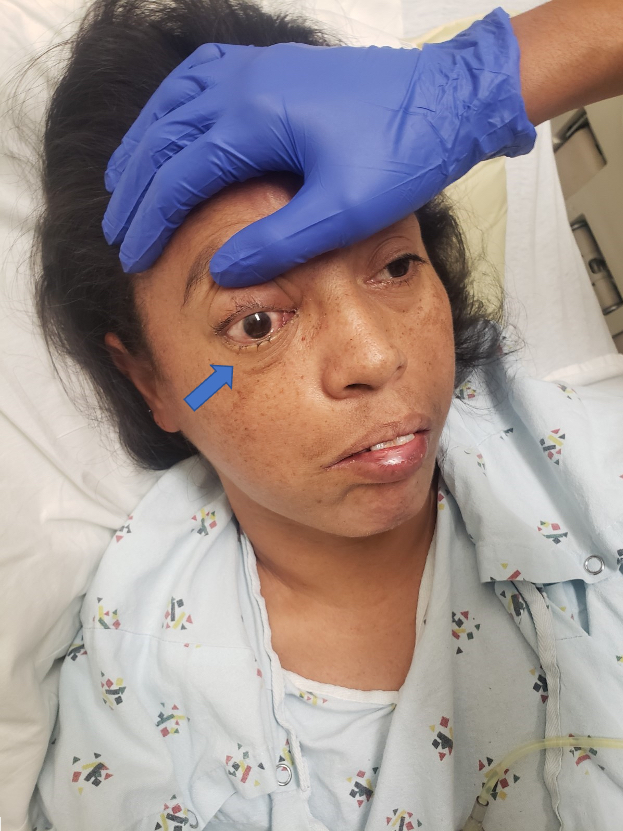

Supplement: Supplementary file 3 [file JETem-8-3-V10-supp3.jpg]

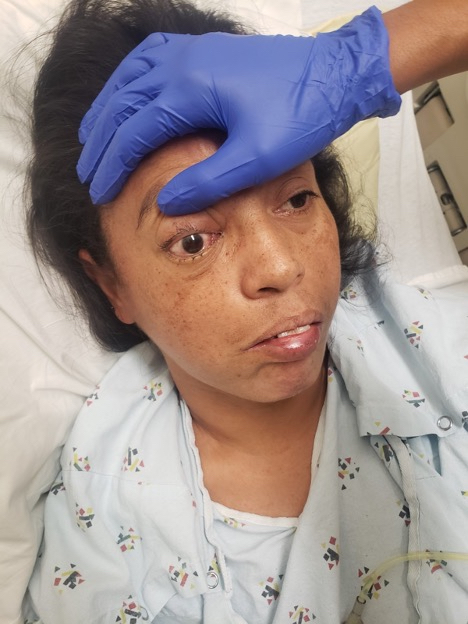

Supplement: Supplementary file 4 [file JETem-8-3-V10-supp4.jpg]

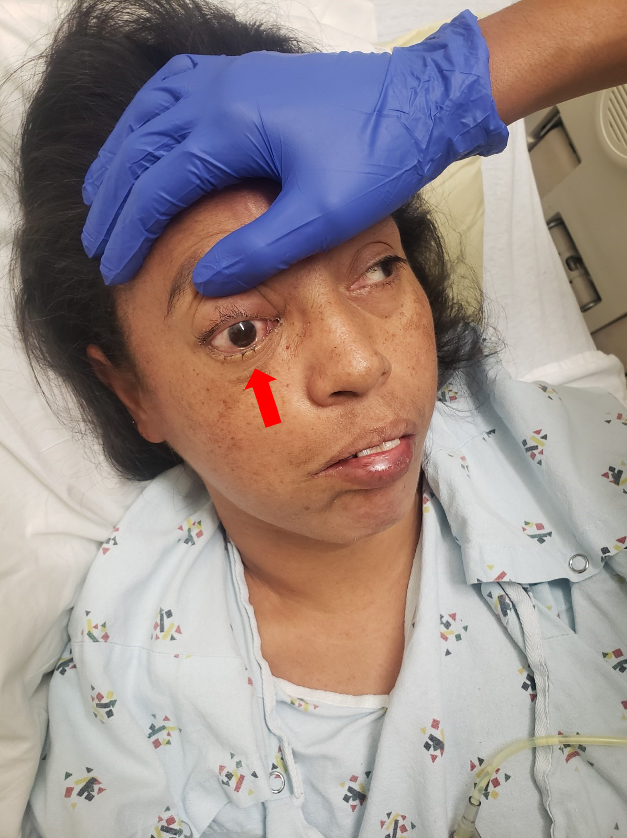

Supplement: Supplementary file 5 [file JETem-8-3-V10-supp5.jpg]

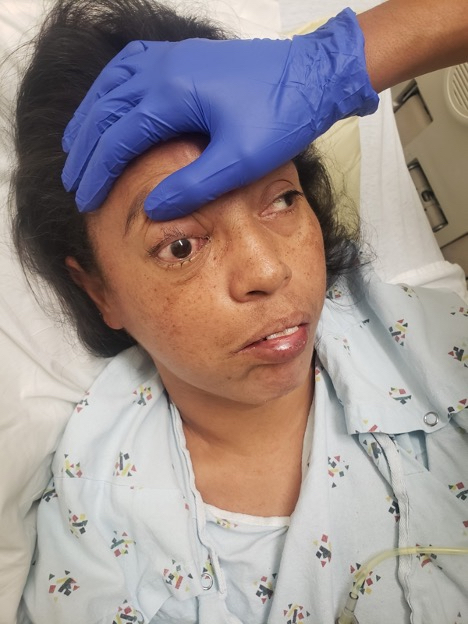

Supplement: Supplementary file 6 [file JETem-8-3-V10-supp6.jpg]

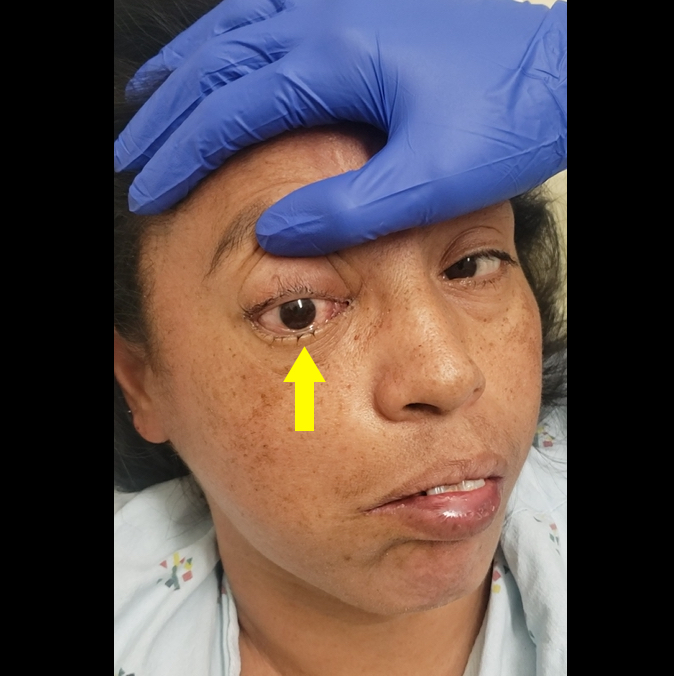

Supplement: Supplementary file 7 [file JETem-8-3-V10-supp7.jpg]

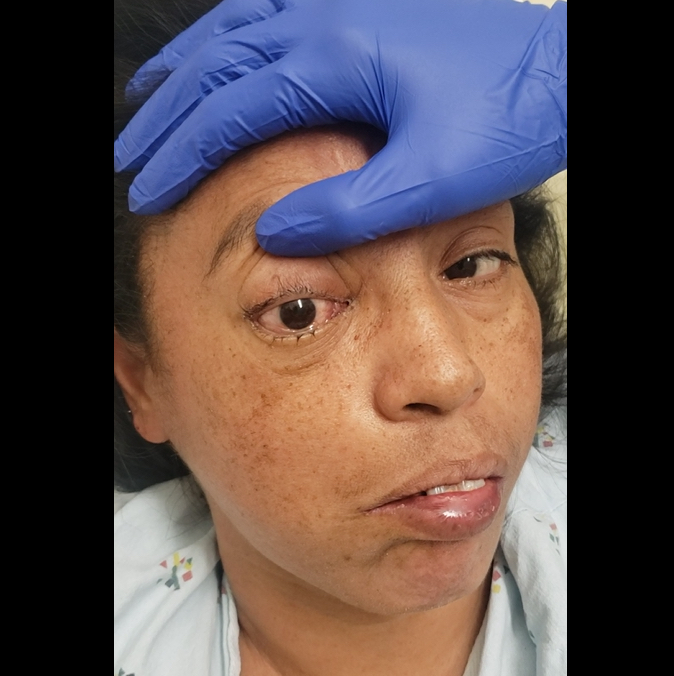

Supplement: Supplementary file 8 [file JETem-8-3-V10-supp8.jpg]
